# Use of Wearable Devices for Peak Oxygen Consumption Measurement in Clinical Cardiology: Case Report and Literature Review

**DOI:** 10.2196/45504

**Published:** 2023-08-15

**Authors:** Gabriella Bayshtok, Shmuel Tiosano, Ariel Furer

**Affiliations:** 1 Sackler School of Medicine Tel Aviv University Tel Aviv Israel; 2 Arrow Program for Medical Research Education Sheba Medical Center Tel Hashomer Ramat Gan Israel; 3 Leviev Heart Center Sheba Medical Center Ramat Gan Israel; 4 Department of Military Medicine Faculty of Medicine The Hebrew University of Jerusalem Jerusalem Israel

**Keywords:** cardiac fitness, cardiac patient, cardiorespiratory fitness, CRF, clinical cardiology, oxygen consumption, peak VO2, smartwatch, wearable device

## Abstract

**Background:**

Oxygen consumption is an important index to evaluate in cardiac patients, particularly those with heart failure, and is measured in the setting of advanced cardiopulmonary exercise testing. However, technological advances now allow for the estimation of this parameter in many consumer and medical-grade wearable devices, making it available for the medical provider at the initial evaluation of patients. We report a case of an apparently healthy male aged 40 years who presented for evaluation due to an Apple Watch (Apple Inc) notification of low cardiac fitness. This alert triggered a thorough workup, revealing a diagnosis of familial nonischemic cardiomyopathy with severely reduced left ventricular systolic function. While the use of wearable devices for the measurement of oxygen consumption and related parameters is promising, further studies are needed for validation.

**Objective:**

The aim of this report is to investigate the potential utility of wearable devices as a screening and risk stratification tool for cardiac fitness for the general population and those with increased cardiovascular risk, particularly through the measurement of peak oxygen consumption (VO_2_). We discuss the possible advantages of measuring oxygen consumption using wearables and propose its integration into routine patient evaluation and follow-up processes. With the current evidence and limitations, we encourage researchers and clinicians to explore bringing wearable devices into clinical practice.

**Methods:**

The case was identified at Sheba Medical Center, and the patient’s cardiac fitness was monitored through an Apple Watch Series 6. The patient underwent a comprehensive cardiac workup following his presentation. Subsequently, we searched the literature for articles relating to the clinical utility of peak VO_2_ monitoring and available wearable devices.

**Results:**

The Apple Watch data provided by the patient demonstrated reduced peak VO_2_, a surrogate index for cardiac fitness, which improved after treatment initiation. A cardiological workup confirmed familial nonischemic cardiomyopathy with severely reduced left ventricular systolic function. A review of the literature revealed the potential clinical benefit of peak VO_2_ monitoring in both cardiac and noncardiac scenarios. Additionally, several devices on the market were identified that could allow for accurate oxygen consumption measurement; however, future studies and approval by the Food and Drug Administration (FDA) are still necessary.

**Conclusions:**

This case report highlights the potential utility of peak VO_2_ measurements by wearable devices for early identification and screening of cardiac fitness for the general population and those at increased risk of cardiovascular disease. The integration of wearable devices into routine patient evaluation may allow for earlier presentation in the diagnostic workflow. Cardiac fitness can be serially measured using the wearable device, allowing for close monitoring of functional capacity parameters. Devices need to be used with caution, and further studies are warranted.

## Introduction

Oxygen consumption has been measured and appreciated in clinical cardiology for decades, most commonly in advanced cardiopulmonary exercise tests (CPETs) in a complex laboratory setup. It is a parameter used to evaluate cardiorespiratory fitness (CRF), thus named peak oxygen consumption (VO_2_), representing an individual’s largest volume of oxygen extracted from inhaled air during efforts [1]. This measurement is affected by age, gender, genetics, underlying medical conditions, and physical activity, especially high-intensity training [2-4]. The routine use of this parameter in the setting of CPET is to assess changes in cardiac capacity following exertional physical activity and serve as a validated prognostic factor in cardiovascular patients. This parameter further allows for the evaluation of the interactions between the cardiac, musculoskeletal, respiratory, and vascular systems [5] and is of great importance due to its association with decreased all-cause mortality [6,7]. Table S1 in Multimedia Appendix 1 [1,8-14] summarizes the definitions and abbreviations of the key terms.

Wearable devices have gained tremendous popularity in recent years and were traditionally divided into consumer products and medical-grade devices. However, these distinctions are rapidly fading as top-selling consumer devices now provide validated and regulatory-approved medical-grade measurements of physiologic parameters, including heart rate and oxygen saturation, using a 1-lead electrocardiogram and photoplethysmography. Companies such as Apple, Garmin, Fitbit, and Samsung distribute wearable devices that allow for validated peak VO_2_ measurements using proprietary algorithms with a combination of heart rate and pace data during exercise compared to a user’s baseline. As of 2018, the Food and Drug Administration (FDA) categorizes the Apple Watch (Apple Inc) as a class II device for over-the-counter use of an electrocardiogram (ECG) and photoplethysmography. These tools allow for the identification of cardiac conditions such as atrial fibrillation [15,16]. In 2020, Apple released a new version of its wearable device that estimates submaximal oxygen consumption; however, further FDA approval and classification are still needed for this utility. During Apple’s peak VO_2_ assessment, various sensors such as a photoplethysmograph, gyroscope, accelerometer, barometer, and GPS are manipulated to maximize the algorithm to estimate peak VO_2_ in the general population. Study participants completed CPETs with treadmills and cycle ergometers while wearing the Apple Watch Series 4, and linear projections estimated peak VO_2_ using age-predicted maximum heart rates in the submaximal range (at least a 30% increase in heart rate). Algorithm predictions were compared with the average of all CPET measurements (at least 6) for each participant. A comparison between the 2 modalities found the Apple Watch to be valid (mean difference of 1.4, SD 4.7), reliable (intraclass correlation coefficient [ICC] of 0.86), and consistent (median of 1.2 and 90th percentile SD per participant of 2.6 on the Apple Watch for those with at least five estimates). The Apple Watch estimates peak VO_2_ with an average error of less than 1 metabolic equivalent (MET) compared to CPETs. Apple Watch peak VO_2_ measurements were found to be more reliable than submaximal treadmill tests (ICC of 0.87 vs 0.75), which are performed if a patient cannot complete a full CPET. Limitations to algorithm performance include pacemakers, medical conditions causing chronotropic incompetence, exercise intolerance, and arrhythmias. Apple supports the development of measuring cardiac fitness and addresses limitations such as rate-limiting medications through algorithm adjustment [17]. Ongoing clinical trials are underway to investigate the use of the Apple Watch to measure cardiopulmonary fitness in ambulatory heart failure patients [18]. Table S2 in Multimedia Appendix 1 [1,8-14] includes a list of other devices used for this purpose and details the evidence available for their validation.

The technological advancement of wearable devices, combined with their fast distribution and adoption by many, sets the stage for using this data in the preemptive screening of wide populations, including apparently healthy individuals. To demonstrate this concept (illustrated in Multimedia Appendix 2), we present the case of a young and previously fit individual without previous medical conditions who was alerted to a decline in his cardiac fitness index from his Apple Watch despite being asymptomatic. We describe the initial patient workup until the final diagnosis and then review the body of knowledge available investigating oxygen consumption and its clinical uses. Finally, we review current data on technologies and devices in this field and the potential clinical benefits of routinely using such technologies.

## Methods

The Apple Watch Series 6 was used for cardiac fitness measurements. The data were provided by the patient from the Apple Health app.

### Case

**Figure 1:**
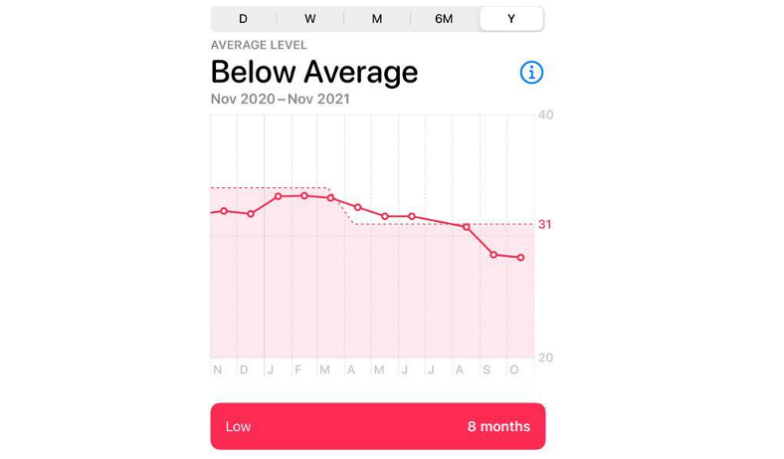
Screenshot of the patient’s Apple Watch cardiac fitness index from November 2020 to October 2021 that triggered the initial cardiac evaluation.

A male patient aged 40 years presented following a notification from his Apple Watch indicating low cardiac fitness. The patient had been using the Apple Watch since November 2020 and received the alert in October 2021 during a trip abroad. His device revealed a progressive decline in cardiac fitness index, as shown in Figure 1. The patient had no known previous medical history, hospitalizations, or medical treatment. He was fit and engaged in high-intensity workouts several times a week. Other than occasional palpitations, he reported being asymptomatic during the months before this alert. He reported no history of smoking or use of illicit substances and no family history of cardiovascular disease, including sudden death or ischemic disease. At the time of presentation, however, he reported that his sister had been undergoing a cardiac workup at the same time due to suspected peripartum cardiomyopathy following supraventricular tachycardia (SVT) after delivery. Her echocardiography exam suggested mild globally reduced left ventricle (LV) systolic function and an ejection fraction (EF) of 45%.

Initial workup included a stress echocardiography exam demonstrating reduced global LV systolic function and an LV EF of 20% (visually estimated) without valvular malformations or regional wall abnormalities. Following these results, the patient was hospitalized and underwent a comprehensive workup, including repeated echocardiography, a 24-holter exam, and cardiac magnetic resonance imaging (MRI). Repeat echocardiography demonstrated a mildly dilated LV, severe diffuse global LV dysfunction with an EF of 23%, grade I diastolic dysfunction, a right ventricle with normal size and function, a normal left atrium, an aortic valve with minimal regurgitation, mild mitral regurgitation, and minimal tricuspid regurgitation with normal systolic pulmonary pressure. The rest of the exam was within normal limits. Cardiac MRI demonstrated a dilated LV with an LV end-diastolic diameter of 63 mm. There were no signs of late gadolinium enhancement. Normal T1 mapping was up to 1150 milliseconds and T2 mapping was up to 52 milliseconds (with normal values up to 50 milliseconds); the measured LV EF was 47%. The next step in the cardiomyopathy workup was genetic testing, which revealed a titin mutation (*TTN*, exon 326, c.86116C>T [p.Arg28706*]).

**Figure 2:**
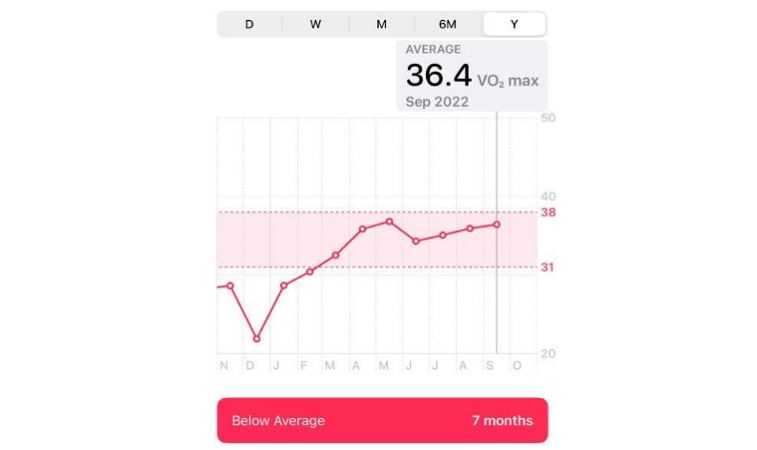
Screenshot of the patient’s Apple Watch cardiac fitness index from November 2021 to September 2022 after treatment initiation.

During hospitalization, the patient was started on a β-blocker, mineralocorticoid receptor antagonist, an angiotensin II receptor blocker, and a sodium-glucose transport protein 2 inhibitor. After hospitalization, he began a cardiac rehabilitation program with no reports of symptoms on exertion; the New York Heart Association classification was 1. Additional echocardiography in December 2021 demonstrated a LV EF of 35%. Figure 2 depicts an increase in the patient’s cardiac fitness index from November 2021 to September 2022 after treatment initiation.

### Ethics Approval

The participant has provided written informed consent. All patient data has been deidentified.

## Discussion

### Overview

In recent years, we have witnessed growth in the accessibility of oxygen consumption measurement and its potential uses in clinical cardiology. Peak VO_2_ helps establish a reference for a patient’s cardiac fitness and provides insight into risk stratification for disease status, cardiac rehabilitation, and perioperative status. Oxygen consumption improvement has been shown to confer a survival benefit and minimize disease progression [19], and therefore, measurement of this parameter is useful for both one-time assessment and routine follow-up. The continuous feedback of peak VO_2_ through wearable devices may allow for patient engagement in health monitoring, promote lifestyle changes, and guide clinical decision-making [20]. This case of an asymptomatic male with low peak VO_2_ due to underlying cardiomyopathy demonstrates the advantages of wearable devices as a screening tool in healthy and at-risk adults, as well as the ability to monitor cardiac fitness after treatment initiation. Many other cardiac and noncardiac pathologies may be detected earlier due to their correlation to changes in peak VO_2_. We discuss the various pathologies and clinical scenarios that relate to this index below.

Oxygen consumption has been shown to be decreased in various cardiac conditions, including heart failure (HF), ischemic heart disease (IHD), atrial fibrillation (AF), valvular disease, cardiomyopathy, hypertension, and other pathologies [21-23]. HF patients often experience decreased quality of life with restriction of basic activities due to dyspnea and exercise intolerance, with peak VO_2_ being a well-studied estimator of functional capacity among this patient population. A study of chronic systolic HF patients showed that a 6% increase in peak VO_2_ was associated with improved clinical outcomes, including all-cause mortality and hospitalizations [24]. Additional studies found that increased peak VO_2_ due to systemic and skeletal muscle adaptations from high-intensity training is associated with the preservation of ejection fraction and prevention of LV remodeling [25]. From a hemodynamic standpoint, oxygen consumption has a strong linear correlation with cardiac output [26], making peak VO_2_ tracking a surrogate marker for cardiac output estimation, which is essential among HF patients. Another study found that better cardiac fitness in midlife (median age 49 years) is associated with a decreased risk of developing HF and subsequent hospitalization later in life, independent of other cardiac or noncardiac risk factors (1 MET increase was associated with a 17% risk reduction) [27]. Interestingly, in a study with 63 patients with chronic AF who underwent cardioversion to sinus rhythm, peak VO_2_ was monitored before the procedure and 1 month after. Peak VO_2_ max after the procedure significantly increased, suggesting that low peak VO_2_ accompanies AF, perhaps related to tachycardia-induced cardiomyopathy [28]. Hypertension also warrants oxygen consumption monitoring, as a prospective study reported that those in a lower VO_2_ max group were almost 2 times more at risk of developing hypertension and that striving for a higher VO_2_ max may be protective against hypertension [29].

Peak VO_2_ measurement is also relevant in noncardiac diseases. Studies have shown that increased oxygen consumption is associated with a lower risk of developing metabolic syndromes such as diabetes through several mechanisms. First, exercise, as reflected by improved VO_2_, builds muscle that uses and removes glucose from the blood, while other reports discuss the potential role of oxidative pathway regulation in mitochondria as a potential linker between oxygen consumption and metabolic risk [30,31]. CRF has been found to have an inverse relationship with metabolic syndrome in males and females, with waist circumference being the strongest predicting factor [32]. Additional studies report that improved CRF is as effective as statin therapy in lowering the mortality risk in patients with dyslipidemia [33]. The benefit of increased peak VO_2_ can also be seen among patients with chronic obstructive pulmonary disease (COPD) and is associated with reduced all-cause mortality [1]. Further investigation of the relationship between peak VO_2_ and other chronic medical conditions is warranted, as low peak VO_2_ is associated with ongoing disease progression in patients with rheumatoid arthritis, as estimated by inflammatory markers and subjective assessment. Cardiovascular risk factors linked to rheumatoid arthritis, such as atherosclerosis and changes in fat and muscle distribution, were decreased among patients with improved aerobic capacity [34]. Moreover, it is hypothesized that a higher peak VO_2_ is protective against brain pathologies such as stroke, depression, and dementia [35-37]. To investigate the relationship between cardiorespiratory fitness and stroke risk, a study examined 16,878 asymptomatic men aged between 40 and 87 years whose fitness was followed over 10 years, reporting that a higher VO_2_ was associated with a 68% lower risk of stroke death when compared to those with a low VO_2_, and a moderate VO_2_ was associated with a 63% decrease [35]. As discussed here, there is a widespread need for oxygen consumption evaluation among pathologies extending from cardiology, especially because many conditions may not produce symptoms until later in the disease course.

Cardiac rehabilitation is perhaps the best proof of practice for following peak VO_2_ to reduce all-cause mortality and hospital admissions. For patients with acute coronary syndrome (ACS) or surgical interventions, cardiac rehabilitation is imperative for secondary prevention. Future directions for rehabilitation intend to maximize the incorporation of exercise to achieve greater aerobic capacity, which can be monitored through changes in peak VO_2_ [38]. Additionally, reports comment on the advantage of objective physical activity and prognostic factor monitoring for personalized feedback, which relies less on patient recollection [39]. Peak VO_2_ also allows for assessing patients’ readiness for cardiovascular and noncardiovascular surgery, with studies demonstrating its association with postoperative complications and mortality in procedures such as gastrointestinal and vascular surgeries, hepatic transplantation, lung tumor resection, and coronary artery bypass grafting [40]. Thus, this dynamic parameter may be considered in the preoperative assessment, leading to improved decision-making and outcomes. A unique role for peak VO_2_ measurement is evaluating a patient’s need or readiness for heart transplantation. A study with 181 HF patients reported that the actuarial 1- and 2-year survival of the 89 patients who achieved a VO_2_ equal to or <50% of predicted peak VO_2_ was 74% and 43%, respectively, compared with 98% and 90% in the 92 patients who achieved >50% predicted peak VO_2_ [41]. Other studies have investigated VO_2_’s role in triaging for cardiac transplantation and proposed a peak VO_2_ greater than 14 ml/min/kg as an appropriate cutoff value [42]. Further validation studies are needed to assess whether oxygen consumption measurement is a suitable marker for heart transplantation.

As with this patient’s case, most individuals with cardiomyopathy accompanied by decreased left ventricular systolic dysfunction are asymptomatic until the development of advanced disease [43]. It is difficult to estimate the number needed to screen to prevent cardiac events as the data concerning the validity of wearable peak VO_2_ is preliminary and the true scope of the asymptomatic cardiac patients’ burden is uncertain. However, serial peak VO_2_ measurements have been shown to be advantageous in certain populations, such as those with congenital heart diseases. In a study with 1375 adult cyanotic and noncyanotic heart disease patients followed for 10 years while undergoing serial CPETs, the combination of peak VO_2_ and heart rate reserve provided strong prognostic insight for mortality [44]. Another study in adults with a Fontan circulation who completed at least two maximal CPETs found that a decrease in peak VO_2_ is a predictor of death or transplant, regardless of initial peak VO_2_ [45]. These studies suggest that peak VO_2_ measurements may be a useful prognostic marker for heart disease burden due to congenital heart defects, and perhaps in individuals similar to our patient after further studies are performed. We suggest there may be a place for using peak VO_2_ data among both apparently healthy adults and those in high-risk populations as a first warning sign, triggering further workup. With most screening tools, there is a concern that false-positive data will cause unnecessary, costly, and, at times, hazardous workups. We believe that a more precise role for this tool will be only determined after a careful assessment is performed with robust scientific methodology and sufficient statistical power.

As reviewed here, a large body of evidence suggests the potential role of integrating peak VO_2_ into various clinical scenarios. The option to access data routinely and continuously using a wearable device necessitates reconsidering peak VO_2_’s ideal place in clinical settings. Wearable devices can at least partially replace a patient’s need to seek a CPET, which remains a logistically complex exam mostly limited to medical institutions, resulting in inconvenience for patients and poor accessibility. Reports recognize the potential of wearables for primary and secondary prevention but also identify barriers and limitations such as cost, data security, and false-positive results leading to unnecessary intervention and subsequent burden on the health care system [46]. The heterogeneity of methods used to collect data, along with the continuous device updates, similarly presents challenges for standardization [39]. Other reports have introduced a guide for integrating wearable devices into cardiovascular care with considerations for incorporating data into electronic medical records, staff education, index cutoffs, and frequency of review [47]. Further limitations of wearable devices include the inability to measure peak carbon dioxide production (VCO_2_) and anaerobic threshold values, which serve as additional prognostic factors measured during CPETs. Peak VCO_2_ is the amount of carbon dioxide exhaled from the body over time and reflects exercise capacity, whereas the anaerobic threshold is the point of substantially increased minute ventilation relative to VO_2_ and represents a rise in lactic acid production. The anaerobic threshold suggests the functional capacity of HF patients, with a lower measurement indicating decreased capacity. It can also distinguish between cardiac and noncardiac conditions, with fatigue before the threshold point being less indicative of a cardiac condition [48].

We demonstrated that wearable devices could streamline the process from the onset of disease manifestation to therapeutic intervention. Indeed, a potential new trend that medical providers will need to address is the presentation of peak VO_2_ or parallel indices by the patient through wearables at an early phase or as the trigger for a diagnostic workflow before other modalities, especially among HF patients. Moreover, routine peak VO_2_ measurement in HF patients allows for another objective variable to assess, in addition to standard parameters such as weight, blood pressure, and heart rate, which addresses the issue of sparse, unbiased indices by broadening the opportunity for concrete data collection. Measuring this index continuously and remotely, which is not relevant for CPETs and other conventional methods that calculate peak VO_2_, allows patients to present earlier in the usual diagnostic workflow. After diagnosis and treatment initiation, peak VO_2_ can be monitored using the wearable device, as demonstrated in this patient’s case. Health care providers and patients can be more informed about cardiac fitness during a treatment or rehabilitation regimen and accordingly adjust interventions while preventing clinical exacerbations. There is potential to improve clinical outcomes through enhanced monitoring of functional capacity parameters and cardiac fitness; however, devices should be used cautiously and only for adult populations in the appropriate clinical setting to prevent adverse use. Additional prospective studies are needed to enable this change while being supported with scientific evidence and an official classification as a medical-grade device. With the exponentially growing distribution of wearable devices, it may be less of a challenge to conduct these studies and obtain policy-changing evidence in a short period of time.

### Conclusions

This report demonstrates the potential utility of routine peak VO_2_ measurement for screening cardiac and noncardiac diseases to elucidate therapeutic options for patients in an accelerated timeframe and leverage widespread technology to initiate early interventions. Peak VO_2_ provides a glimpse into risk stratification, rehabilitation, and perioperative care. Greater awareness of cardiorespiratory fitness among apparently healthy adults and those with high cardiovascular risk is needed and may result in impactful interventions.

## Appendix

Multimedia Appendix 1 Table S1: Definitions and abbreviations of key terms; Table S2: Comparison of devices on the market providing an estimation of oxygen consumption or surrogate indices.

Multimedia Appendix 2 Graphical abstract. CPET: cardiopulmonary exercise testing; ECG: electrocardiogram; VO2: oxygen consumption.

## Abbreviations

ACS: acute coronary syndrome
AF: atrial fibrillation
COPD: chronic obstructive pulmonary disease
CPET: cardiopulmonary exercise test
CRF: cardiorespiratory fitness
ECG: electrocardiogram
EF: ejection fraction
FDA: Food and Drug Administration
HF: heart failure
ICC: intraclass correlation coefficient
IHD: ischemic heart disease
LV: left ventricle
MET: metabolic equivalent
MRI: magnetic resonance imaging
SVT: supraventricular tachycardia
VCO_2_: carbon dioxide production
VO_2_: oxygen consumption

## Data Availability

All data generated for this publication are included in the manuscript.
