# Supplementary material for: Use of Wearable Devices for Peak Oxygen Consumption Measurement in Clinical Cardiology: Case Report and Literature Review
Source: Interact J Med Res. 2023 Aug 15;12:e45504. doi: 10.2196/45504 (PMC10466150; doi:10.2196/45504)
Supplement: Multimedia Appendix 1 [file ijmr_v12i1e45504_app1.docx]

**Table S1**. Definitions and abbreviations of key terms [1].

| Term | Abbreviation | Definition |
| --- | --- | --- |
| Oxygen consumption | VO_2_ | Rate of oxygen extracted from inhaled air by the body during physical exertion. |
| Maximal VO_2_ | VO_2_ max | The maximum achievable rate of oxygen consumption during physical exertion [1]. VO_2_ max can be estimated using the formula:  15 $\times$ (HR_max_/HR_rest_) [43]. |
| Peak VO_2_ | VO_2_ peak | The highest amount of oxygen extracted from inhaled air during peak exercise. |
| Cardiorespiratory fitness | CRF | The ability of the respiratory and circulatory systems to supply oxygen to the body for energy during efforts. |
| Cardio-pulmonary exercise testing | CPET | The measurement of respiratory gas exchange to assess cardiac capacity changes following maximal exercise. |

**Table S2.** Comparison of devices on the market providing an estimation of oxygen

consumption or surrogate indices.

| Device | Evidence | Reference |
| --- | --- | --- |
| Garmin | The Garmin watch (Garmin, Kansas, USA) has been found to measure VO_2_ max with 95% accuracy in a study performed with 79 runners with an estimated error of less than 3.5ml/kg/min, measured against VO_2_ max values in laboratory tests [44]. Another study assessing the validity of peak VO_2_ with the Garmin fitness watch, Forerunner 920XT, found no significant difference between the watch compared to measurements obtained via open-circuit spirometry [45]. Garmin Fenix S6 demonstrated a valid 30-second VO_2_ max estimate compared to lab testing [46]. The above models require heart rate data for valid peak VO_2_ estimation. | [44,45,46] |
| Fitbit | Fitbit generated valid VO_2_ max measurements using the smartwatch's GPS, heart rate, and pace. After users complete a run of at least 10 minutes, accurate VO_2_ max estimation is generated compared to a graded exercise test conducted on a treadmill using specialized equipment. Of the 65 healthy adults in the study, there was a significant association between the Fitbit CRF (cardiorespiratory fitness) and VO_2_ max, with a mean absolute percentage error of less than 10% [47]. | [47] |
| Samsung Galaxy watch | The Samsung Galaxy watch provides peak VO_2_ readings while running with high intensity for at least 20 minutes with GPS; however, additional validation studies are needed [48]. | [48] |
| Other | Patches, chest straps, and hand-held devices have also been tested for this aim [49,50] but further validation of the devices is needed in the future. | [49,50] |
