# Supplementary figures and images for: Use of Wearable Devices for Peak Oxygen Consumption Measurement in Clinical Cardiology: Case Report and Literature Review
Source: Interact J Med Res. 2023 Aug 15;12:e45504. doi: 10.2196/45504 (PMC10466150; doi:10.2196/45504)

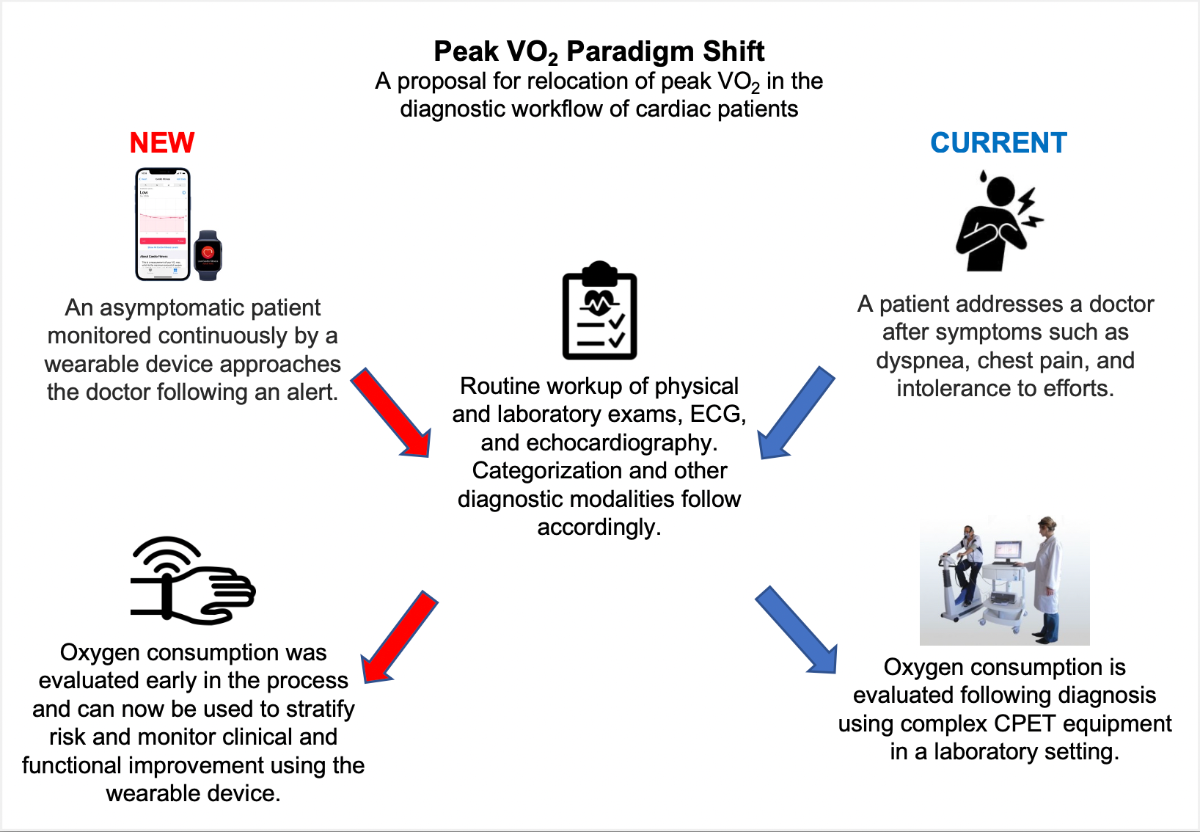

Supplement: Multimedia Appendix 2 [file ijmr_v12i1e45504_app2.png]
